# Cryptic Interactions in a Marine Microcosm: Sponges Shape the Microbiome of a Resident Goby

**DOI:** 10.1111/mec.70523

**Published:** 2026-08-20

**Authors:** Ingrid Vasconcellos Bunholi, Abbie Affleck, Kallen Johnson, Reynaldo Ortega, Christopher J. Freeman, Theresa Rueger, Jordan M. Casey

**Affiliations:** ^1^ The University of Texas at Austin Marine Science Institute Port Aransas Texas USA; ^2^ Dove Marine Laboratory, School of Natural and Environmental Science Newcastle University Newcastle upon Tyne UK; ^3^ University of Belize Belmopan Belize; ^4^ Department of Biology College of Charleston Charleston South Carolina USA

**Keywords:** 16S rRNA sequencing, coral reefs, cryptobenthic fish, fish gut microbiome, skin microbiome, species interactions

## Abstract

Microbial symbionts influence ecological and evolutionary processes in their multicellular hosts; yet they remain poorly characterised in marine systems. Sponges host exceptionally diverse microbiomes, potentially providing rich microbial reservoirs for their residents. The tusked goby ( *Risor ruber* ), an obligate sponge‐dweller, and its sponge hosts provide an ideal model to investigate the causes and consequences of shared microbial taxa in host–resident systems. Using 16S rRNA sequencing, we characterised the microbiomes of *R. ruber* skin and gut, their sponge hosts, a non‐obligate, sponge‐associated goby ( *Elacatinus prochilos* ) and seawater. We quantified microbial overlap among hosts, examined ontogenetic patterns of shared microbiomes across goby body size and assessed the putative ecological benefits of shared microbial taxa for *R. ruber* . We reveal more than 500 microbial ASVs shared between *R. ruber* and their sponge hosts. The unique microbial overlap between *R. ruber* and their sponge hosts far exceeded overlap with *E. prochilos* and seawater. Shared taxa are putatively linked to nutrient acquisition, vitamin biosynthesis and pathogen defence, suggesting potential benefits for *R. ruber* from inhabiting microbially rich sponges*.* The proportion of the shared microbiome between *R. ruber* gut and sponges declines with goby body size, which suggests that the goby may undergo ontogenetic changes in diet or gut development*.* Thus, sponges provide more than shelter: they may function as microbial reservoirs and nutritional sources for residents. These findings underscore the need to protect microbially rich habitats and further study host–resident microbial interactions in marine systems and beyond.

## 1 Introduction

Species interactions are fundamental to the origin and maintenance of biodiversity across ecosystems (Bairey et al. 2016; Chesson 2000; Connolly and Roughgarden 1999). Ecologists often focus on simple pairwise interactions; however, species interactions frequently occur in high‐order combinations, where the interaction between two species is modulated by the presence or absence of others (Melián et al. 2009; Mougi and Kondoh 2012). Biotic interactions occur across a wide range of ecological scales, from entire food webs (Berlow et al. 2009; Casey et al. 2019; Horn et al. 2019) to cryptic bipartite interactions such as metabolite exchange between autotrophic microbes and dinoflagellates (Koch and Schmid‐Hempel 2011; Millette et al. 2018). The degree of specialisation and variation in interaction strength can profoundly influence ecological dynamics and even drive species diversification (Blüthgen et al. 2007; Hembry et al. 2014). For example, bromeliad‐associated frogs complete their entire life cycle within bromeliads, offering nutritional benefits to the plant in exchange for shelter and a reservoir of food (Romero et al. 2010; Sabagh et al. 2017). To understand the ecological and evolutionary implications of such specialised relationships, it is essential to first identify all interacting partners, including the cryptic ones and clarify the nature of their association.

Among cryptic interactions, uncovering the interactions between multicellular hosts and their microbial symbionts represents a rapidly growing frontier (Medina and Sachs 2010). These ubiquitous host‐microbe associations, ranging from the most complex animals (e.g., humans and their gut microbiome) to the most basal ones (e.g., Scleractinian corals and the microbiome of their exodermal mucus), are shaped by two broad mechanisms: host‐intrinsic factors and environmental factors. Host‐intrinsic factors involve genetics (Griffiths et al. 2019), vertical transmission from parent to offspring (Schmiedová et al. 2020) and evolutionary history like phylosymbiosis (Pollock et al. 2018). Environmental factors include a range of biotic and abiotic factors, including geographic location (Swierts et al. 2018), diet (Degregori et al. 2024) and physical contact (Kremer and Huigens 2011). Host–microbe interactions are key components of ecological and evolutionary dynamics, but we know much less about interactions that involve multiple partners, such as the role of microorganisms in eukaryotic symbioses (Fronk and Sachs 2022). For example, microbes contribute to bee–angiosperm mutualisms (Steffan et al. 2024), influence host–parasite dynamics in bumble bees (Koch and Schmid‐Hempel 2011), mediate resistance to pathogens in amphibians (Rebollar et al. 2018) and shape pathogen transmission in insect vectors (Weiss and Aksoy 2011).

Much less is known about microbial interactions in marine systems, but host‐associated, benthic organisms represent an ideal focal group to examine shared microbes among eukaryotes. On coral reefs, invertebrates such as corals and sponges dominate the benthos and are often covered by nutrient and microbe‐rich mucus, which mediates species interactions, such as nutrient acquisition by predators like fishes (Pratte et al. 2018; Semmler et al. 2025). Host‐associated microbes are often shaped by close interspecific associations, especially in the case of spatially constrained environments and long‐term relationships between species (Émie et al. 2021; Sarkar et al. 2020). For example, in clownfish–anemone–microbiome symbiosis, clownfish obtain nutrients from their host anemone, the anemone's microbiome recycles these nutrients and the clownfish microbiome is shaped by physical contact with the anemone (Alan Verde et al. 2015; Pratte et al. 2018). Similarly, in sponges, polychaetas acquire host‐derived microbes via feeding, enriching rare bacterial taxa in their own gut microbiomes (Turon et al. 2019). Symbiotic microorganisms in sponges can comprise up to 35% of the total holobiont volume (Hentschel et al. 2012) and cryptobenthic reef fishes that live in close association with sponges can exhibit microbial overlap with their invertebrate hosts (Xavier et al. 2019).

*Risor ruber* , an obligate sponge‐dwelling goby with limited dispersal potential, presents an exceptional model system to explore microbiome convergence between closely associated eukaryotes and deepen our understanding of interactions between mobile organisms and their benthic hosts (Coppock et al. 2022; Wang et al. 2024). *Risor ruber* inhabits the internal canals of massive and tubular sponges across the western Atlantic Ocean and has evolved morphological adaptations associated with permanent life within sponge canals, including specialised dentition that facilitates movement and foraging within confined canal networks (Tyler and Böhlke 1968). This close association with sponges purportedly provides *R. ruber* with food and shelter (Rocha et al. 2000; Tyler and Böhlke 1968). In addition, the sponge hosts of *R. ruber* include high microbial abundance (HMA) sponge genera, such as *Xestospongia*, *Aplysina* and *Ircinia* (Freeman et al. 2021; Lesser et al. 2022), making them rich microbial reservoirs for their residents. However, whether these sponge‐associated microbial communities are reflected in the microbiome of *R. ruber* remains unknown. Characterising this relationship may reveal previously overlooked microbial associations and provide a foundation for evaluating their role in goby nutrition and health.

Here, we examine whether the close association between a sponge host and resident goby facilitates a shared microbiome and identify the causes and consequences of microbial overlap. In addition, to provide broader ecological context, we assess how the sponge host‐associated microbiome shapes the skin and gut microbiomes of *R. ruber* compared to a non‐obligate, sponge‐associated goby ( *Elacatinus prochilos* ) that inhabits coral and sponge surfaces. We then propose potential mechanisms driving these associations and explore the potential benefits of microbial enrichment for *R. ruber* . Our study thus examines how obligate residence within a microbially rich sponge is reflected in the microbiome of a resident goby and provides a foundation for evaluating the ecological significance of cryptic microbial associations.

## 2 Materials and Methods

### 2.1 Goby and Sponge Collections

**FIGURE 1:**
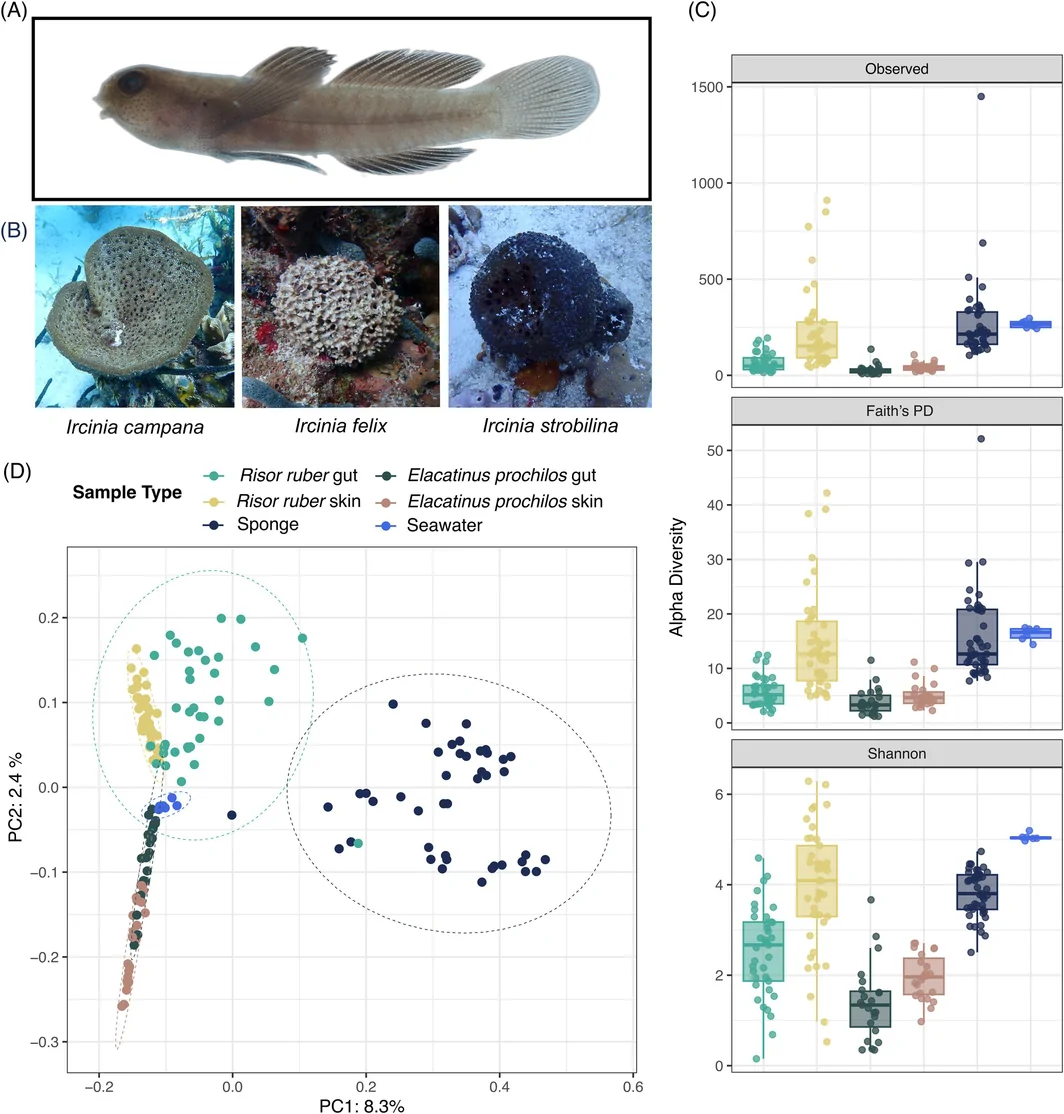
Focal species and their associated microbial diversity. (A) *Risor ruber* (tusked goby; total length = 19.3 mm) collected from a shallow reef at 5.4 m depth. (B) Three sponge species, including *Ircinia campana* collected at 6 m, *Ircinia felix* at 5.6 m and *Ircinia strobilina* at 8.5 m. (C) Alpha diversity metrics for each sample type, showing microbial richness (Observed), phylogenetic diversity (Faith's PD) and evenness (Shannon). Each point represents a sample, while boxplots summarise quantiles. Points are coloured by sample type. (D) Unweighted UniFrac principal coordinates analysis (PCoA) illustrating the microbial community composition of *R. ruber* and *E. prochilos* skin and gut, sponge and seawater. Points are coloured by sample type and ellipses represent 95% confidence intervals.

When sponges were occupied by *Risor ruber* , we collected *R. ruber* and sponge samples from three species of *Ircinia* sponges (*Ircinia campana, Ircinia felix* and *Ircinia strobilina* ) across four patch reefs near Carrie Bow Cay, Belize in July 2023 (Figure 1a,b and Figure S1). All collections were made at shallow depths (< 10 m) via SCUBA. Overall, we collected 45 *R. ruber* specimens and 45 sponge samples ( *I. campana* = 16, *I. felix* = 27 and *I. strobilina* = 2). All specimens were collected opportunistically, with roving divers targeting any of the three *Ircinia s*ponges that could potentially host *R. ruber* (following (Wang et al. 2024)). After finding a target sponge, we photographed the individual with a ruler for scale and recorded the depth. A tag was added near the base of each sponge to avoid resampling the same individual. All tags were removed and discarded at the end of the study. A mixture of 5:1 ethanol to clove oil solution was sprayed around and inside the sponge, targeting the excurrent pores, to anaesthetise any fish inside the sponge. After waiting for approximately 30 s for the anaesthetic to take effect, we squeezed and wafted the *R. ruber* out of the sponge and placed them into a pre‐labelled, unique Ziplock bag with seawater. All divers wore gloves and avoided touching the fish during collections. To pair microbiome data from the fish and sponge hosts, we also collected small tissue samples from each sponge where *R. ruber* specimens were collected. We collected sponge samples with scissors and placed each sample into an individual Whirl‐Pak with seawater. Once the specimens were brought to the surface, we placed them on ice and brought them to the Carrie Bow Cay Field Station for further processing.

### 2.2 Microbiome Sample Processing and Sequencing

In the laboratory, we prepared skin and gut microbiome samples from each *R. ruber* individual. We sterilised all tools and equipment with a 10% bleach solution. The skin microbiome was sampled by swabbing one entire side of the body back and forth three times using sterile cotton swabs (Cotton Tipped Applicators, Dynarex, USA). We dissected each goby using a dissecting microscope. Given the tiny size of the fish (average: 1.6 cm), we removed the entire gastrointestinal tract of each fish for our gut microbiome samples. Skin swabs and gastrointestinal tracts were each placed into sterile 2 mL tubes filled with preservation buffer (QIAGEN DNeasy PowerSoil Pro Kit C1 buffer). Sponge microbiome samples were obtained by cutting cross sections in the sponge, including both the inner and outer tissue regions and preserving the cross sections in 95% ethanol. *Ircinia campana* , *Ircinia felix* and *Ircinia strobilina* were identified based on underwater photographs using The Sponge Guide (https://spongeguide.uncw.edu/) and (Kelly and Thacker 2021).

We extracted DNA from skin, gut and sponge samples at the University of Texas Marine Science Institute using a QIAGEN DNeasy PowerSoil Pro Kit following the manufacturer's protocol with a few modifications. For the skin and gut samples, before sample homogenisation, samples were incubated at 65°C for 10 min and 95°C for 10 min. For the sponge samples, we first cut our sponge cross sections into smaller fragments, then we incubated samples at 65°C for 30 min and lengthened the homogenisation to 15 min. We included negative controls to account for contamination during extraction. DNA extracts were quantified using a Qubit Fluorometer and diluted to equal concentrations (~4 ng/μL) prior to PCR amplification.

We amplified the V4 16S rRNA gene region using 515F and 806R primers (Caporaso et al. 2011) following the Earth Microbiome Project protocol. Each PCR reaction was carried out in a 25 μL volume containing 5 μL of 1 μM forward and reverse primer, 12.5 μL of 2× KAPA HiFi HotStart ReadyMix (KAPA Biosystems) and 2.5 μL of DNA template (~12.5 ng). Thermocycler conditions were as follows: an initial denaturation at 95°C for 3 min; 8 cycles of 95°C for 30 s, 55°C for 30 s and 72°C for 30 s; followed by a final extension at 72°C for 5 min. Amplification success was verified via electrophoresis on a 1% agarose gel. PCR products were purified using AMPure XP Beads (Beckman Coulter). Library preparation was performed at the Hubbard Center for Genome Studies at the University of New Hampshire using a dual‐indexing approach with the Nextera XT Index Kit (Illumina). Indexing PCR reactions were carried out in 50 μL volumes containing 5 μL each of Index 1 (i7) and Index 2 (i5), 25 μL of 2× KAPA HiFi HotStart ReadyMix, 10 μL PCR‐grade water and 5 μL DNA template. Thermocycling conditions matched the initial amplification: 95°C for 3 min; 8 cycles of 95°C for 30 s, 55°C for 30 s and 72°C for 30 s; and a final extension at 72°C for 5 min. Final libraries were cleaned with AMPure XP Beads and quantified using a Qubit Fluorometer. Equimolar pooling was performed and the combined libraries were normalised to 4 nM and sequenced on an Illumina NovaSeq 6000 using a 500‐cycle NovaSeq Reagent Kit (Illumina).

### 2.3 Bioinformatic Processing

We processed the sequences with QIIME2 (v.2024.5) for quality control, denoising, amplicon sequence variant (ASV) identification and taxonomic assignment. To provide ecological context for microbial associations in sponge‐dwelling gobies, we re‐analysed two publicly available 16S rRNA datasets: (i) skin and gut microbiomes from 23 *Elacatinus prochilos* individuals from Barbados (Xavier et al. 2019—SRA BioProject PRJNA527319) and (ii) six seawater samples from Carrie Bow Cay, Belize (Gantt et al. 2019—SRA BioProject SRP142647). These datasets were used as a comparative framework among an obligate, sponge‐dwelling goby ( *R. ruber* ), a non‐obligate, sponge‐associated goby ( *E. prochilos* ) and the environment (seawater samples). Because the *E. prochilos* samples were collected at a distinct location and time and the seawater samples were collected at a different time, these samples do not represent a fully matched control.

We performed quality control on demultiplexed pair‐end sequences ( *R. ruber* gut, *R. ruber* skin, sponge, *E. prochilos* gut, *E. prochilos* skin and seawater) using FastQC (Brown et al. 2017). Primers were removed with the *cutadapt trim‐paired* plugin in QIIME2 (Bolyen et al. 2019; Martin 2011). Reads were denoised and merged separately for each sample type using the *dada2 denoise‐paired* plugin (Callahan et al. 2016). Truncation lengths for forward and reverse reads were chosen based on interactive quality plots generated by the *qiime demux summarize* plugin to remove low‐quality tails. Reads were filtered based on the default expected error threshold (maxEE = 2) and chimeras were removed using the consensus method (default in DADA2). Following denoising, we merged the datasets for taxonomic assignment. Posterior taxonomy assignment was done with the *feature‐classifier classify‐consensus‐vsearch* plugin in QIIME2 using the SILVA database (Quast et al. 2012). ASV filtration and the curation of taxonomic assignments were completed in R version 4.5 (R Core Team 2025). Taxonomic ranks were assigned using the following percent identity thresholds: species and genus assignments required ≥ 97% identity, family to class required ≥ 85% and phylum required ≥ 70%. ASVs were annotated only to the highest rank meeting these pre‐defined confidence thresholds.

We removed singleton ASVs, ASVs detected in negative controls and reads assigned to Eukaryota, Archaea, chloroplasts and mitochondria. Samples containing fewer than 5000 total reads were excluded as low‐depth sequencing libraries. This quality control filtration step retained 183 samples, including 43 *R. ruber* skin, 45 *R. ruber* gut, 43 sponge, 23 *E. prochilos* skin, 23 *E. prochilos* gut and six seawater samples (Table S1). Across these samples, 32 *R. ruber* skin, *R. ruber* gut and sponge samples have matching microbiome data (i.e., *R. ruber* skin and gut samples from the same individual, as well as its host sponge sample; Table S2). Across the retained samples, we obtained 25,609,598 sequence reads (mean = 139,943 per sample) and produced a feature table containing 73,153 ASVs, of which 59,373 could not be confidently assigned at the domain level. Because the initial depth threshold was applied before restricting the feature table to bacterial ASVs, sequencing depth was recalculated and samples containing fewer than 1000 bacterial reads were excluded. In addition, ASVs with zero abundance after sample removal and ASVs not assigned to Bacteria at the domain level were removed (Table S3). After domain‐level ASV filtering, the dataset contained 13,716 ASVs and 173 samples ( *R. ruber* skin = 41, *R. ruber* gut = 37, Sponge = 43, *E. prochilos* skin = 23, *E. prochilos* gut = 23 and seawater = 6).

### 2.4 Data Analysis

Alpha diversity was calculated at the ASV level using Shannon diversity, Faith's phylogenetic diversity (PD) and observed ASV richness using the R packages *phyloseq* (McMurdie and Holmes 2013) and *picante* (Kembel et al. 2010). To assess differences in alpha diversity across sample types, we applied generalised linear models (GLMs) based on the distribution of each diversity metric. The predictor variable was ‘sample type’ categorical; *R. ruber* skin, *R. ruber* gut, sponge, *E. prochilos* skin, *E. prochilos* gut and seawater and model residuals were visually inspected to confirm assumptions of each distribution. We did not observe significant differences in microbial diversity across the three *Ircinia* sponge species; therefore, we combined all sponge species for downstream analyses (Figure S2; Table S4).

To assess ASV‐level differences in bacterial community composition among sample types, beta‐diversity was estimated using an unweighted UniFrac distance matrix (Lozupone and Knight 2005) and visualised with a principal coordinate analysis (PCoA). We employed the UniFrac metric because it captures microbial diversity at multiple taxonomic scales and is better suited for broad comparative analyses (Lozupone and Knight 2005). We examined differences across sample types using permutational analysis of variance (PERMANOVA—999 permutations) using the *adonis* function and homogeneity of multivariate dispersion using the *betadisper* function in the R package *vegan* (Oksanen et al. 2025).

To examine microbial overlap between *R. ruber* and sponge samples, we removed ASVs assigned to the phylum Cyanobacteria because they are not part of the endogenous skin or gut fish microbiome. Using this filtered dataset, we converted the ASV count table to presence‐absence to quantify unique and overlapping ASVs. Then, to quantify overlap between fish and sponge microbiomes, for each *R. ruber* gut and skin sample, we calculated the percentage of bacterial reads assigned to ASVs that also occurred in at least one sponge sample. Finally, we used a null model framework adapted from Wang et al. (2024), based on randomised binary interaction matrices, to statistically evaluate whether ASV‐level microbial overlap between *R. ruber* and sponge samples differed from random expectations. For this null model analysis, the dataset was rarefied to 5000 reads per sample to reduce the influence of uneven sequencing depth on pairwise ASV sharing. Observed binary interaction matrices were generated with links defined as fish‐sponge pairs sharing at least five ASVs. In each null matrix, each interaction cell was presence (1) or absence (0) with equal probability (*p* = 0.5). Each null model was simulated 999 times and observed link counts were compared to the resulting distributions to compute *p*‐values. Finally, we performed sensitivity analyses to the defined thresholds by (1) summarising pairwise overlap across thresholds of ≥ 1, ≥ 2, ≥ 3, ≥ 5 and ≥ 10 shared ASVs and (2) repeating the analysis using fixed‐link probabilities of 0.1, 0.2 and 0.3 (Tables S5 and S6).

After evaluating ASV‐level microbial overlap between *R. ruber* and sponge samples, we examined genus‐level overlap in comparison to *E. prochilos* and seawater samples. We retained genera detected in at least 5% of sponge samples to provide an inclusive view of genus‐level associations while limiting overlap of genera detected in only one or two sponge samples. We visualised overlap among groups using an UpSet plot from the R package UPSETR version 1.4.0 (Conway et al. 2017). We only visualised overlap that included sponges and at least one additional sample type.

To identify the key shared microbial genera between *R. ruber* and sponge microbiomes, we applied a more restrictive filtering scheme to highlight prevalent shared genera. We calculated relative read abundances across genera that were present in at least 10% of the sponges, were not present in a co‐occurring goby ( *E. prochilos* skin or gut) or environmental samples (seawater) and had more than 50 reads in the *R. ruber* skin, *R. ruber* gut and sponge samples. We performed sensitivity analyses on the two thresholds: the percent occurrence in sponges and number of read counts. After analysing various thresholds, we ascertained that the selected thresholds were appropriate to retain biologically relevant shared genera, while excluding rare detections or low sequence counts (Table S7). For visualisation, relative read abundance was calculated within the pooled *R. ruber* tissue and sponge datasets.

Finally, we investigated specific microbial associations between *R. ruber* and their sponge hosts. First, we assessed whether sponge microbial community composition differed among *Ircinia* species using unweighted UniFrac distances. Differences in community composition were visualised using principal coordinates analysis and tested using permutational multivariate analysis of variance (PERMANOVA). We then examined whether *R. ruber* individuals shared more bacterial ASVs with the sponge host from which they were collected compared to other sponges by calculating pairwise relative abundance overlaps between each fish and sponge microbiome. Finally, we assessed whether overlap between *R. ruber* skin or gut microbiomes and their corresponding host sponge varied with fish body size. Bayesian beta‐regression models with a logit link function were fitted for *R. ruber* skin and gut separately using the R package *brms* (Bürkner 2017) to model the relative abundance of shared taxa as a function of *R. ruber* total length (mm). Only matched fish‐host sponge pairs with overlap values greater than zero and less than one were included. We opted for a Bayesian approach because the paired microbiome dataset was relatively small and right‐skewed, which can cause issues in frequentist beta regressions. We used default weakly informative priors. The mean and credible intervals were estimated based on posterior values derived from five chains of 10,000 iterations, including a warm‐up of 5000 iterations. To visualise predicted trends, we generated posterior draws of the expected mean response across the range of observed body sizes using the function *add_epred_draws* from the R package *tidybayes* (Kay 2024). These analyses were repeated after combining ASVs at the genus level. All the statistical analyses were performed in R v4.5.0 (R Core Team 2025).

## 3 Results

### 3.1 Microbial Diversity Across Fishes, Sponges and Seawater

Microbial diversity significantly differs across sample types, with *R. ruber* skin, sponge and seawater samples exhibiting higher alpha diversity compared to the other sample types, followed by *R. ruber* gut samples (Figure 1c; Table S8). In addition, at the ASV‐level, we detected distinct patterns in bacterial communities across sample types (PERMANOVA: pseudo‐*F*_(5,167)_ = 5.133, *p* = 0.001; Figure 1d; Table S9). Notably, we found a separation between *Ircinia* sponges and fish‐associated ASV‐level microbial communities. Multivariate dispersion also differed significantly among sample types (PERMDISP: *F*_(5,167)_ = 70.551, *p* = 0.001; Table S8), indicating that the observed pattern reflects differences in community composition and within‐group heterogeneity. At the genus level, fish‐associated microbiomes had greater overlap, while sponge samples remained distinct from fish samples (Figure S2; Table S9).

### 3.2 The Shared Microbiome Between *Risor ruber* and Its Sponge Host

**FIGURE 2:**
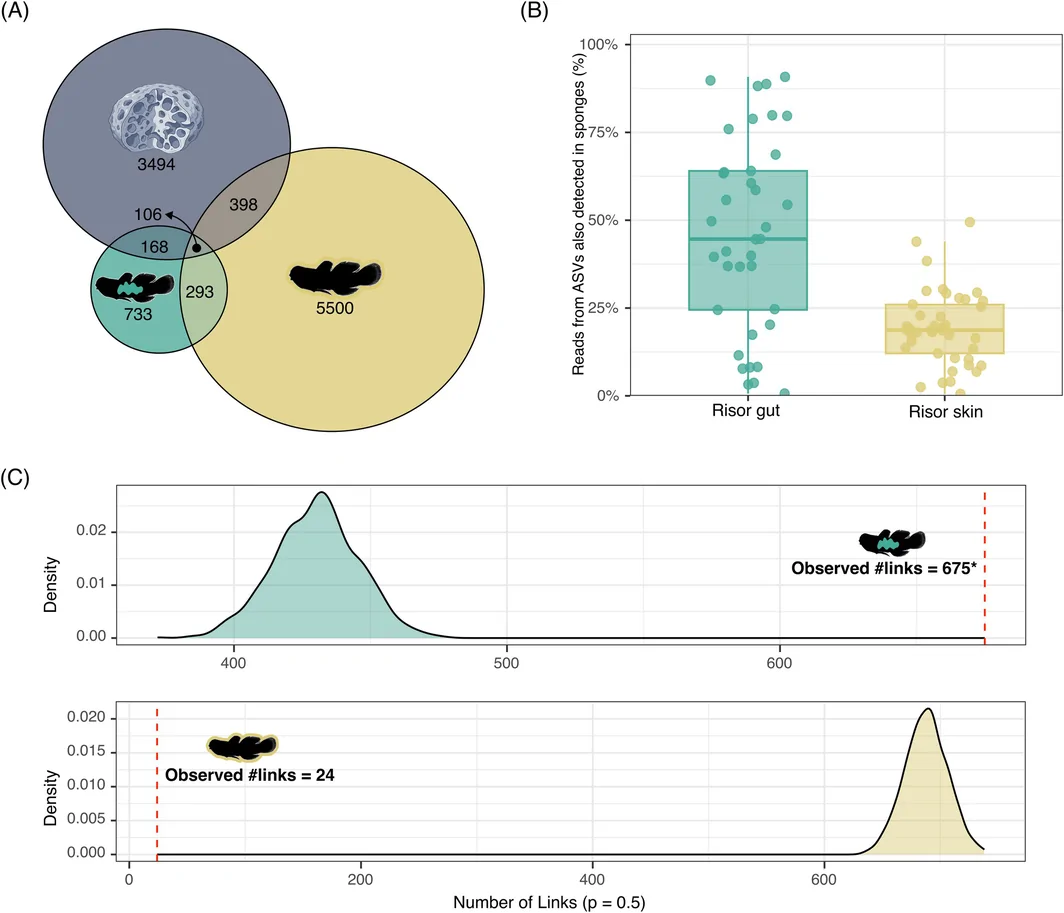
ASV‐level microbial overlap between *Risor ruber* and sponge microbiomes. (A) Venn diagram showing unique and shared microbial ASVs among *R. ruber* gut, *R. ruber* skin and sponges. (B) Percent of sequence reads in *R. ruber* gut and skin assigned to ASVs also detected in sponges. Points represent individual samples, while boxes show the median and interquartile range. (C) Null‐model analysis of *Risor*‐sponge links sharing at least five ASVs. Density plots represent the null distribution of expected ASV‐level links across 999 sample null models with fixed link probability (*p* = 0.5). Red dashed lines indicate observed links in the empirical data. Values to the right of the density plot indicate more links than expected by chance, while links to the left indicate fewer links than expected. Sponge icon was created in BioRender. Bunholi, IV. (2026) https://BioRender.com/ihcyebp.

At the ASV‐level, we observed substantial overlap between *R. ruber* and sponge microbiomes (Figure 2a). A total of 106 ASVs occurred in all the sample types. *Risor ruber* skin and sponge microbiomes shared a total of 504 ASVs, including 398 ASVs detected exclusively in these two sample types, whereas *R. ruber* gut and sponge microbiomes shared 274 ASVs, including 168 ASVs detected only in gut and sponge microbiomes (Figure 2a). Despite sharing fewer ASVs with sponges in absolute numbers, *R. ruber* gut microbiome contained a larger proportion of reads assigned to ASVs that also occurred in sponges compared to *R. ruber* skin microbiome. These co‐occurring ASVs consisted of 46.2% ± 27.8% of reads in *R. ruber* gut microbiomes and 19.3% ± 10.7% of reads in *R. ruber* skin microbiomes, with substantial among‐individual variation, particularly in *R. ruber* gut microbiomes (Figure 2C; Table S10).

Compared to a null model, the observed ASV‐level overlap between *R. ruber* gut and sponge microbiomes was higher than expected by chance ( *R. ruber* gut‐sponge = 675 links versus null mea*n* = 431 links; *p* < 0.001), indicating non‐random microbial overlap between *R. ruber* guts and their sponge host (Figure 2c). In contrast, *R. ruber* skin‐sponge overlap was lower than expected under the same null model ( *R. ruber* skin‐sponge = 24 links versus null mean = 688 links). Thus, although many ASVs were shared between *R. ruber* skin and sponges, at the pooled group level, ASVs co‐occurring with sponges were more consistently detected in the gut than the skin.

**FIGURE 3:**
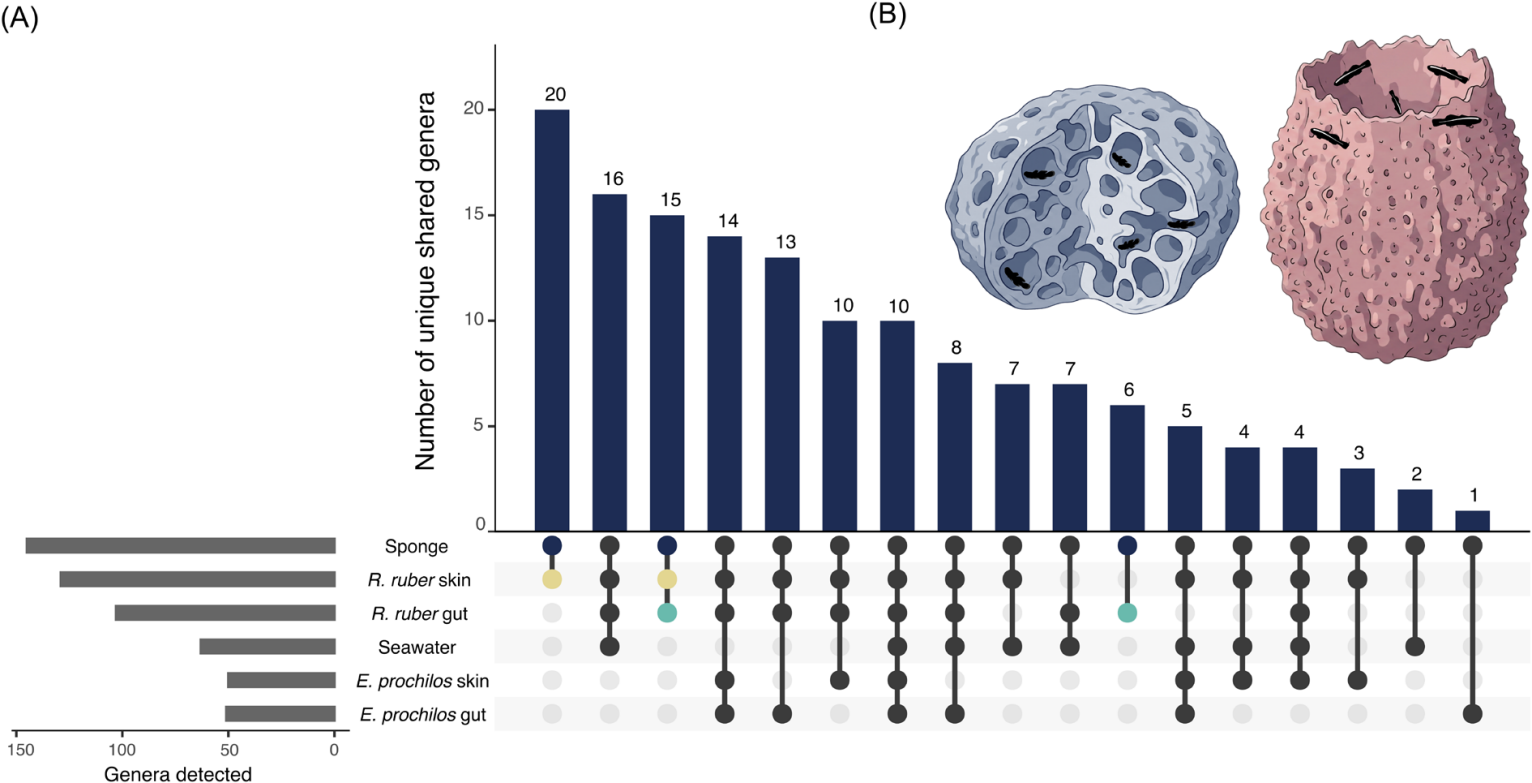
Genus‐level microbial overlap among *Risor ruber* gut and skin and their sponge host. (A) The number of unique microbial genera shared across the different samples. *R. ruber* ‐sponge overlap are represented by yellow ( *R. ruber* skin‐sponge) and green ( *R. ruber* gut‐sponge) points. (B) Schematic representation of the obligate sponge‐dwelling goby *R. ruber* within *Ircinia* sponge canals and the non‐obligate, sponge‐associated *Elacatinus prochilos* on the surface of *Xestospongia muta* . Created in BioRender. Bunholi, IV. (2026) https://BioRender.com/ihcyebp.

At the genus‐level, we included *Elacatinus prochilos* and seawater samples in the comparative framework. We detected the greatest overlap between *R. ruber* skin and sponge (*n* = 20), *R. ruber* skin, gut, seawater and sponge (*n* = 16) and *R. ruber* skin, gut and sponge (*n* = 15). In an analysis of all intersections between sponges and the *R. ruber* microbiome, we detected 41 exclusively shared microbial genera. In contrast, only one genus was exclusively shared between sponges and *E. prochilos* guts, while two were shared exclusively between sponges and seawater (Figure 3a).

**FIGURE 4:**
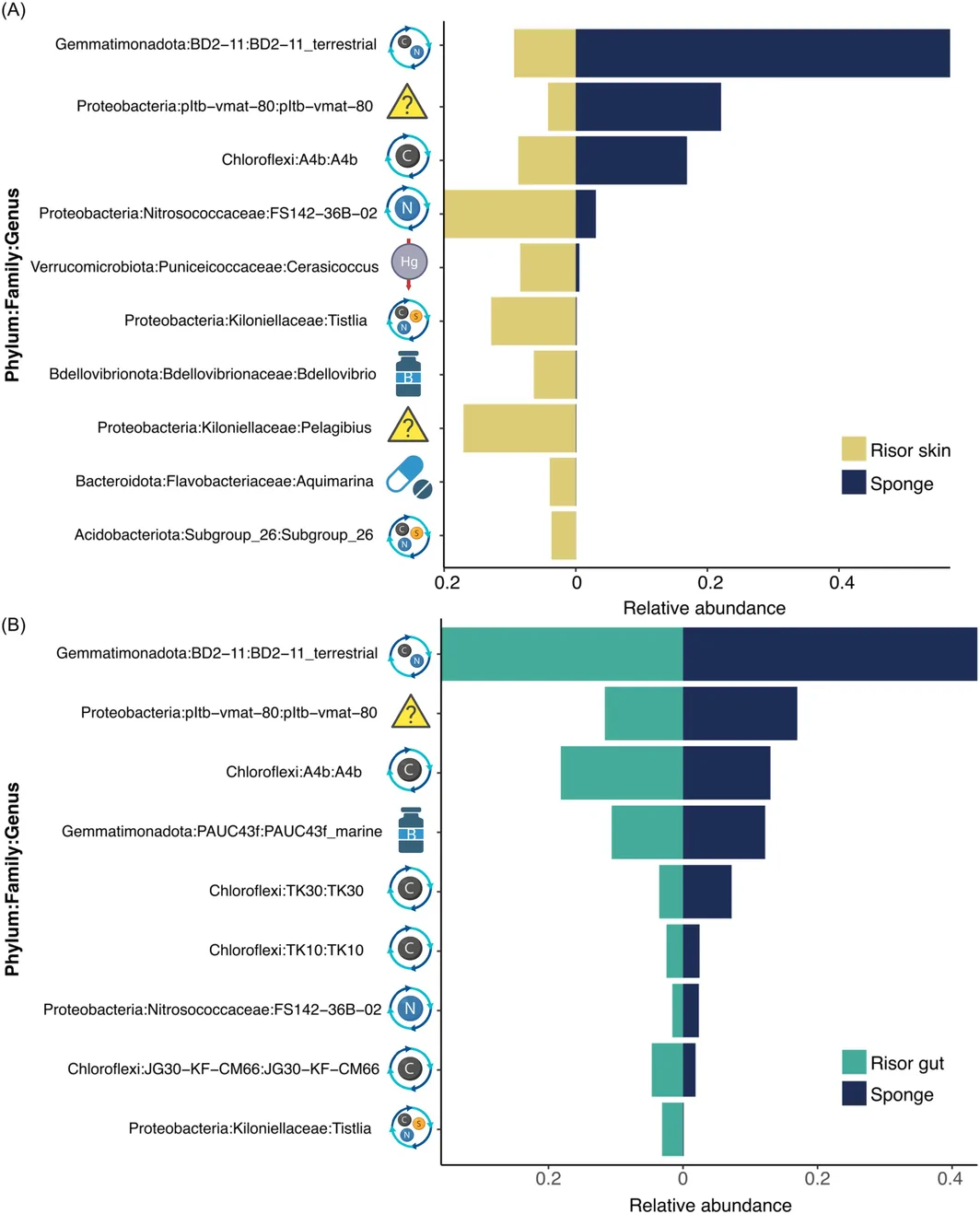
Key shared microbial genera between *Risor ruber* and sponges. (A, B) The relative abundance of microbial genera shared between *R. ruber* skin (A) or gut (B) and sponges, based on all genera present in at least 10% of sponges, absent from a co‐occurring goby ( *E. prochilos* skin and gut) and seawater samples and containing more than 50 reads in both *R. ruber* and sponge samples. Icons following the microbial taxa represent inferred microbial functions based on the literature (see Table S7 for more details). Created in BioRender. Bunholi, IV. (2026) https://BioRender.com/0d1qk8w.

Using the most restrictive filtering to identify commonly shared genera (genera present at 10% of the sponges and more than 50 reads), we identified 22 key microbial genera consistently shared between *R. ruber* and sponge microbiomes: 12 were shared between *R. ruber* skin and sponges and 10 between *R. ruber* gut and sponges (Table S11). Collectively, these microbial genera represented 26% of the pooled sponge sequence reads, 0.77% of pooled *R. ruber* skin reads and 0.76% of pooled *R. ruber* gut reads. The shared microbial genera were primarily assigned to the phyla Proteobacteria (*n* = 4), Chloroflexi (*n* = 4) and Gemmatimonadota (*n* = 2) (Figure 4a,b). Based on a literature review, purported functions associated with these taxa include nutrient cycling (e.g., *BD2‐11_terrestrial_group*, *A4b*, *FS142‐36B‐02*, *Tistlia*, *Subgroup_26*, *TK30*, *TK10* and *JG30‐KF‐CM66*), vitamin production (e.g., *Bdellovibrio and PAUC43f_marine_benthic_group*), mercury reduction (e.g., *Cerasicoccus*) and antimicrobial activity (e.g., *Aquimarina*). The most abundant shared taxon was *BD2‐11_terrestrial_group* (phylum Gemmatimonadota), which is often associated with nutrient cycling (Table S11).

### 3.3 Potential Mechanisms Shaping the Shared *Risor ruber* ‐Sponge Microbiome

**FIGURE 5:**
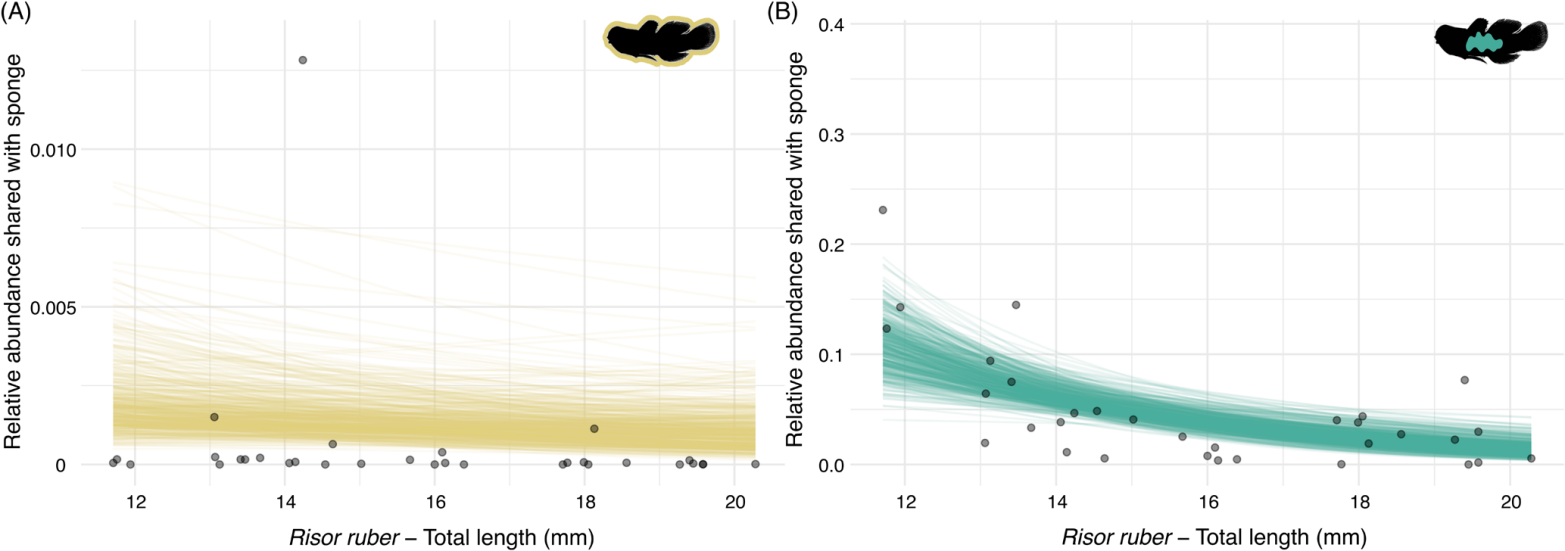
The relationship between *R. ruber* body size and the goby‐sponge shared microbiome. *R. ruber* total length (mm) and the relative abundance of microbial genera shared between *R. ruber* skin (A) or gut (B) and sponge samples based on Bayesian beta regression models. Black points represent each individual fish–sponge pair. Lines depict posterior draws of the expected mean.

At the ASV and genus level, neither *R. ruber* skin nor gut microbiomes had greater overlap with their corresponding host sponge compared to their neighbouring sponges (Figures S3a,b and S4a,b). We further examined whether the overlap between each *R. ruber* microbiome and its corresponding host sponge varied with fish body size. At the ASV level, we found a negative relationship between *R. ruber* body size and *R. ruber* gut‐sponge microbial overlap (posterior mean = −0.25, 95% CI: −0.39 to −0.11), indicating a higher occurrence of shared microbes between the guts of smaller *R. ruber* individuals and their sponge hosts. We did not detect a notable relationship between *R. ruber* body size and the shared microbiome between *R. ruber* skin and their sponge hosts (posterior mean = −0.07, 95% CI: −0.21 to 0.07) (Figure 5a,b). At the genus level, we detected the same pattern (Figure S5a,b; Table S12).

## 4 Discussion

We reveal substantial microbial overlap between *Risor ruber* and their *Ircinia* sponge hosts, with more than 500 ASVs detected in both goby and sponge microbiomes. Notably, at the genus level, the shared microbiome between *R. ruber* and their *Ircinia* sponge hosts is far greater than observed in the non‐obligate, sponge‐associated goby *Elacatinus prochilos* or environmental water samples. These findings suggest that cryptobenthic reef fishes associated with specialised microhabitats may thus derive overlooked microbes from sessile invertebrates, with potential nutritional or immunological benefits. In addition, we identify a link between *R. ruber* body size and the relative abundance of shared microbial taxa between fish guts and sponges, suggesting that this microbial overlap is strongest in smaller individuals and may potentially reflect ontogenetic changes in feeding, immune processes, or gut development. Our findings therefore reveal a previously undocumented dimension of tripartite interactions among sponges, gobies and microbes and provide a foundation for investigating how microbial associations may contribute to the evolution and diversification of cryptobenthic reef fishes.

### 4.1 Sponge Host Shapes Resident Goby Microbiome

Our results suggest that resident cryptobenthic fishes derive some of their microbial diversity from their host. *Risor ruber* skin and gut microbial diversity was significantly higher than a co‐occurring goby, *Elacatinus prochilos* , which inhabits the surfaces of corals and sponges. *Risor ruber* presumably resides deep within the narrow oscula (excurrent openings) of sponges after settlement (Tyler and Böhlke 1968). In addition, *R. ruber* exhibits morphological adaptations, such as specialised dentition, that facilitate movement and assist with gripping the sponge's internal passages (Coppock et al. 2022; Tyler and Böhlke 1968). *Risor ruber* individuals that inhabit *Ircinia* sponges exhibit distinct morphological traits, such as a higher maximum number of scales in a row, as compared to *R. ruber* inhabiting other sponge genera, further demonstrating finely‐tuned microhabitat specialisation (Wang et al. 2024). The combination of high dependency, spatial fidelity and morphological specialisation of *R. ruber* may facilitate consistent microbial acquisition from the sponge environment, potentially contributing to its more diverse microbiome that mirrors its sponge host.

Although persistent exposure to the sponge host may contribute to the microbiome of *R. ruber* , its phylogenetic history also shapes the microbiome. The skin and gut microbiomes of *R. ruber* cluster more closely with the skin and gut microbiomes of *E. prochilos* than their sponge host microbiomes. This separation confirms that fish microbiome is also structured by organism‐specific factors, indicating host filtering that works beyond environmental acquisition. This provides some evidence of phylosymbiosis: host evolutionary history contributes to microbiome composition, which has been widely observed across taxa, including mammals (e.g., mice and hominids), insects (Brooks et al. 2016) and birds (Kropáčková et al. 2017). However, among coral reef fishes, this pattern is weaker (Chiarello et al. 2018; Degregori et al. 2024). While the gobies *R. ruber* and *E. prochilos* shared broad microbial similarity, their microbiome clusters did not fully overlap and they did not share many unique microbial taxa, suggesting that their shared microbiome did not represent a unique, co‐evolved relationship but a broader microbial community common across reef fishes. Our study does not directly test phylosymbiosis, but the compositional separation between fishes and sponges indicates that persistent environmental exposure operates alongside host‐specific processes in structuring the resident goby microbiome. In addition, the *E. prochilos* and seawater datasets were not fully matched controls, so these comparisons have limitations.

The remarkably strong microbial overlap between *R. ruber* and *Ircinia* sponges further supports this rich, shared microbiome. Similar to this goby, polychaetes that live inside sponges also have a strong microbial association with their sponge hosts, with over half of the bacterial taxa in polychaetes also present in their host sponge microbiome (Turon et al. 2019). By remaining closely associated with its sponge host, *R. ruber* may enhance opportunities for horizontal microbial transmission—a mechanism documented in various systems via species interactions (Grupstra et al. 2022), including feeding (insects and plants; (Gonella et al. 2015)), parasitism (aphids and wasps; (Gehrer and Vorburger 2012)) and predation (fishes and corals; (Grupstra et al. 2021)). Horizontal transmission could also be attributed to the potential ectosymbiont role of *R. ruber* (Coppock et al. 2022).

Although *R. ruber* skin shared a high number of microbial ASVs with sponges, shared ASVs were substantially higher between *R. ruber* gut and sponge microbiomes. Skin may be continuously exposed to a broader diversity of bacteria in the wider environment, whereas a smaller subset of these bacteria may be more strongly represented or selectively retained in the gut (Sylvain et al. 2020). The greater overlap between the gut microbiome of *R. ruber* and sponges may reflect direct microbial acquisition through feeding on sponge tissue, detritus, or other materials circulating in the sponge canal (Tyler and Böhlke 1968; Rocha et al. 2000). A detritivorous feeding strategy would be compatible with life in a detritus‐rich environment, although our data do not directly demonstrate feeding‐mediated acquisition. In line with this hypothesis, over half of the bacterial taxa in polychaete guts were also observed in their host sponge microbiome; yet, different polychaete species feeding on the same sponge retained distinct bacterial communities, suggesting species‐specific filtering by the resident organism (Turon et al. 2019).

Despite the substantial microbial overlap between *R. ruber* and sponge microbiomes, we did not observe specific microbial overlap between *R. ruber* and the individual sponge from which they were collected as compared to other sampled sponges. The observed overlap may instead reflect exposure to a broader *Ircinia*‐associated microbial pool rather than individual‐level specificity (see also (Sylvain and Derome 2017)). Beyond environmental exposure, the persistence of sponge‐associated bacteria within the *R. ruber* microbiome, particularly in the gut, may be influenced by developmental processes, including ontogeny (Li et al. 2017), immune‐system development (Kelly and Salinas 2017) and diet (Degregori et al. 2024). The resulting microbiome may therefore reflect a balance between random environmental acquisition and selective microbial retention from the sponge.

### 4.2 Diet and Ontogeny Contribute to Goby Microbiome

Our results reveal that gut‐sponge microbial overlap varies with body size in *R. ruber* , with smaller individuals exhibiting greater overlap with their host sponge microbiome. In contrast, no comparable relationship was detected for the skin microbiome, suggesting that this size‐associated pattern may be specific to the gut. The rich microbial community of sponges may impact the initial microbial colonisation of the *R. ruber* gut microbiome and sponge cellular detritus (Alexander et al. 2014; De Goeij et al. 2013) may be an important resource for *R. ruber* during early life stages. Gut microbiota are shaped not only by environmental factors but also by intrinsic host traits, particularly diet (Colston and Jackson 2016; Degregori et al. 2024). Ontogeny, which often reflects developmental changes in feeding behaviour and physiology, can play a role in the development of the gut microbiome. However, body size is only a proxy for developmental stage and the relationship observed in this study does not directly demonstrate a dietary shift. The decline in overlap may also reflect ontogenetic changes in morphological and physiological differentiation of the gastrointestinal tract during development, immune function, niche availability and microbial interactions (Llewellyn et al. 2014; Sylvain and Derome 2017; Li et al. 2017; Kelly and Salinas 2017). For example, development can impact host‐mediated microbial filtering, as documented in gibel carp ( *Carassius gibelio* ), where fish development was associated with shifts in gut microbiome composition, gut differentiation and increased microbial niche availability (Li et al. 2017).

Interestingly, the giant bacterium *Epulopiscium*, a cigar‐shaped microbe that can reach up to 0.6 mm in length, was consistently detected in the gut microbiome of *R. ruber* (21.9%) of *R. ruber* gut samples but absent from associated sponge samples. This bacterial genus is known as an intestinal symbiont of herbivorous and detritivorous surgeonfishes, where it contributes to the digestion of complex molecules (Clements et al. 1989). To our knowledge, this is the first report of *Epulopiscium* in the gut microbiota of a cryptobenthic reef fish. The occurrence of such a large symbiont in a fish host with a maximum total length of just 2.5 cm (in our study) is striking and should be further investigated. Its presence in the *R. ruber* gut may further support a detritivorous feeding strategy, as is probably common in cryptobenthic fishes (Brandl et al. 2018). Feeding habits and ontogenetic behaviour shifts are incredibly hard to observe in organisms that remain inside their host after settlement. Our study illustrates how microbiome data can provide insights into the natural history of species with cryptic lifestyles.

In addition, we observed a substantial number of bacterial taxa uniquely shared between *R. ruber* skin and gut microbiomes. This suggests an internal–external microbial connectivity, potentially mediated by excretion and egestion, which can transport gut‐associated microbes to surrounding substrates, where they may be acquired on the skin surface (Chiarello et al. 2019) or from egested faecal pellets (Hakim et al. 2016). Interestingly, beyond shaping the gut microbiome of reef fishes, diet may also play an important role in shaping the skin microbiome via indirect transfer of microbes from faeces to skin or the production of variable surface mucus, as observed in butterflyfishes (Chiarello et al. 2019; Reverter et al. 2017).

The microbial overlap between *R. ruber* and sponges may also have functional importance from the sponge's perspective. By consuming detritus enriched with sponge‐associated microbes, *R. ruber* could help regulate microbial and detritus loads that might otherwise interfere with sponge filtration mechanisms. In this way, *R. ruber* may act as an ectosymbiont, supporting sponge health by feeding on detritus without damaging sponge tissue, as has been observed in caridean shrimps (Ďuriš et al. 2011; Horká et al. 2016).

### 4.3 Key Shared Microbial Taxa and Their Putative Functional Roles

Shared microbiota between *R. ruber* and its sponge hosts include taxa that purportedly perform key ecological functions. In sponges, microbial communities are known to contribute to processes such as immune support, pathogen defence, nutrient acquisition and vitamin synthesis (Hentschel et al. 2012). Microbial symbionts may also increase the resilience of sponges to environmental stressors (Posadas et al. 2022). Because *R. ruber* permanently inhabits these microbe‐rich hosts, it is continuously exposed to bacteria with potentially beneficial traits.

Among the key microbial taxa detected in both *R. ruber* skin or gut and their sponge hosts were bacteria associated with nutrient‐cycling (Chloroflexi, Nitrosococcaceae, Kiloniellaceae and Verrucomicrobiota) and B‐vitamin production (Gemmatimonadota and Bdellovibrionota) (Deeg et al. 2020; Engelberts et al. 2020). The phyla Chloroflexi, Proteobacteria and Acidobacteria form part of the core bacterial community consistently found in high microbial abundance sponges, such as *Ircinia* species (Bayer et al. 2018; Gan et al. 2024; González‐Acosta et al. 2022; Hardoim and Costa 2014). Chloroflexi symbionts are directly linked with carbohydrate degradation and may therefore play an important role in recycling dissolved organic matter (DOM) (Bayer et al. 2018). However, many Chloroflexi lineages remain poorly characterised. For example, *A4b*, *TK30*, *TK10* and *JG30‐KF‐CM66*, some of the main shared taxa between *R. ruber* and the sponge microbiome, have unknown functions in sponges, leaving their ecological role in our study system uncertain. Nevertheless, *A4b* is linked to denitrification processes in wetland environments (Feng et al. 2024).

In addition, Proteobacteria, Gemmatimonadota and Acidobacteriota were also highly represented among taxa detected in both *R. ruber* and its sponge host and have been identified as important contributors to B‐vitamin biosynthesis in *Ircinia ramosa* (Engelberts et al. 2020). Animals cannot synthesise several B vitamins and their cofactors on their own, so sponge‐associated microorganisms could potentially have nutritional relevance to *R. ruber* . Specifically, the *PAUC43f* group within Gemmatimonadota, detected in both *R. ruber* gut and sponge microbiomes, has been implicated as an important thiamine producer (vitamin B1) in marine systems (Aldeguer‐Riquelme et al. 2023). Thiamine deficiency is associated with high early‐life mortality in marine fishes (Harder et al. 2018), making the role of *PAUC43f* worth further investigation. Finally, another shared microbe between *R. ruber* and their sponge hosts was *Aquimarina*, which has been associated with antimicrobial activity against *Vibrio*, a known fish pathogen (Silva et al. 2022). However, these putative functional associations are based on taxonomic identity and previously reported traits. As such, we are uncertain of their functional roles in our study system. Future metagenomic, metatranscriptomic, metabolomic and experimental analyses are required to determine the potential importance of these key shared microbes in the *Risor*‐sponge system (Engelberts et al. 2020; Tringe et al. 2005).

## 5 Conclusion

We documented substantial microbial overlap between a benthic invertebrate host and its resident goby, showing that obligate residence in *Ircinia* sponges is reflected in the skin and gut microbiomes of *Risor ruber* . Beyond providing shelter, these sponges constitute a microbially rich habitat that continuously exposes their residents to diverse sponge‐associated bacteria, including taxa with putative roles in nutrient cycling, vitamin production, immune system support and pathogen defence. This underscores the value of considering tripartite interactions among hosts, residents and microbes as key drivers in ecology and evolution across the tree of life.

Moreover, our findings highlight the ecological importance of sponges as habitat‐forming organisms that provide shelter to numerous reef organisms and may support unseen microbial interactions (Maldonado et al. 2015). Sponges may dominate future coral reefs due to their higher resilience to environmental change relative to reef‐building corals (Bell et al. 2013), yet rising ocean temperatures may also compromise sponge health by suppressing metabolic function and disrupting their microbial communities (Fan et al. 2013; López‐Legentil et al. 2008). Such disruptions could have cascading effects on sponge‐associated fauna like *R. ruber* . This possibility emphasises the need to investigate the effects of environmental change on microbially rich sponges and understudied symbiotic, sponge‐associated networks (Bell et al. 2013; Coppock et al. 2022; Wulff 2006).

## Author Contributions

I.V.B. and J.M.C. conceived the idea and designed the methodology. I.V.B., A.A., K.J., R.O., T.R. and J.M.C. collected the data in the field. C.J.F. contributed to methodological design through discussions and verified sponge species identifications. I.V.B. conducted laboratory and data analyses and led the writing of the manuscript. J.M.C. led the editing process and provided guidance throughout the study. All authors contributed critically to the drafts and gave final approval for publication.

## Funding

Funding was provided by the Natural Environment Research Council (NERC), United Kingdom (grant number NE/X012514/1) and by the University of Texas Marine Science Institute (UTMSI). I.V.B. was supported by the Doctoral Discovery Fellowship in Marine Science from UTMSI.

## Ethics Statement

Fieldwork was conducted under permit 0042‐22 Belize Fisheries Department. Ethical approval was granted by the Newcastle University Animal Welfare and Ethical Review Board Project ID No: 1044 and the University of Texas at Austin IACUC AUP‐2021‐00064.

## Conflicts of Interest

The authors declare no conflicts of interest.

## Supporting information


**Figure S1:** Map of the study sites around Carrie Bow Cay on the Belizean Barrier Reef.
**Figure S2:** Unweighted UniFrac principal coordinates analysis (PCoA) illustrating the genus‐level microbial community composition of *R. ruber* and *E. prochilos* skin and gut, sponge and seawater. Points are coloured by sample types and ellipses represent 95% confidence intervals.
**Figure S3:** Unweighted UniFrac principal coordinates analysis (PCoA) illustrating ASV‐level microbial community composition across four *Ircinia* species. Points are coloured by *Ircinia* species and ellipses represent 95% confidence intervals.
**Figure S4:** The relative abundances of shared microbial ASVs between sponge hosts and *Risor ruber* (A) skin and (B) guts. *R. ruber* individuals are ordered by total length along the *x*‐axis. The diagonals indicate shared microbial relative abundances with the sponge individual from which each fish was collected.
**Figure S5:** The relative abundances of shared microbial genera between sponge hosts and *Risor ruber* (A) skin and (B) guts. *Risor ruber* individuals are ordered by total length along the *x*‐axis. The diagonals indicate shared microbial relative abundances with the sponge individual from which each fish was collected.
**Figure S6:** The relationship between *R. ruber* body size and the goby‐sponge shared microbiome (genus level). *R. ruber* total length (mm) and the relative abundance of microbial genera shared between *R. ruber* skin (A) or gut (B) and sponge samples based on Bayesian beta regression models. Black points represent each individual fish–sponge pair. Lines depict posterior draws of the expected mean.

**Table S1:** Number of samples and data source across sample types after filtration.
**Table S2:** Sponge samples with matching *Risor ruber* skin and gut samples collected from the same sponge.
**Table S3:** Number of amplicon sequence variants (ASVs) after main filtrations before data analysis.
**Table S4:** Beta diversity metrics across sponge species at the genus level.
**Table S5:** Rarefied pairwise ASV overlap between *R. ruber* and sponge samples across alternative shared‐ASV link thresholds.
**Table S6:** Sensitivity of the fixed‐probability null model across alternative link probabilities.
**Table S7:** Sensitivity analysis of prevalence and read‐count thresholds used to identify shared microbial genera.
**Table S8:** Alpha‐diversity differences among sample types in the filtered bacterial ASV dataset.
**Table S9:** Beta‐diversity differences among sample types at ASV and genus levels based on unweighted UniFrac distances.
**Table S10:** Representation of bacterial ASVs also detected in sponge samples within *Risor ruber* gut and skin microbiomes.
**Table S11:** Abundance of key shared bacteria and their putative functionality.
**Table S12:** Posterior estimates are from Bayesian beta‐regression models with a logit link, fitted as overlap ~ total length.

## Data Availability

All related metadata and code are available on GitHub https://github.com/Bunholi/Risor_Sponge_Microbiome. Raw sequence reads are deposited to NCBI SRA BioSample Project PRJNA1333385 under accession numbers from SAMN51794804 to SAMN51794936. This research was conducted in the Caribbean in compliance with relevant national regulations and consistent with the principles of the Convention on Biological Diversity and the Nagoya Protocol. Collaborations with regional researchers ensured that local ecological knowledge and context informed study design, fieldwork and data interpretation. Student co‐authors from a local academic institution contributed to specimen collection and identification and were included to promote capacity building and strengthen regional expertise in marine science. Research findings will be made publicly available through open‐access databases, contributing to both local and global understanding of host‐resident interactions in marine systems.
