# Supplementary material for: Cryptic Interactions in a Marine Microcosm: Sponges Shape the Microbiome of a Resident Goby
Source: Mol Ecol. 2026 Aug 20;35(16):e70523. doi: 10.1111/mec.70523 (PMC13492098; doi:10.1111/mec.70523)
Supplement: Supplementary file 1 [file MEC-35-e70523-s002.docx]

**Supplemental Information for:**

***Cryptic interactions in a marine microcosm: sponges shape the microbiome of a resident goby***

Ingrid Vasconcellos Bunholi^1^, Abbie Affleck^2^, Kallen Johnson^3^, Reynaldo Ortega^3^, Christopher J. Freeman^4^, Theresa Rueger^2^, Jordan M. Casey^1^

Supplementary Figures

**Figure S1** Map of the study sites around Carrie Bow Cay on the Belizean Barrier Reef.

**Figure S2** – Unweighted UniFrac principal coordinates analysis (PCoA) illustrating the genus-level microbial community composition of *R. ruber* and *E. prochilos* skin and gut, sponge, and seawater. Points are colored by sample types, and ellipses represent 95% confidence intervals.

**Figure S3** – Unweighted UniFrac principal coordinates analysis (PCoA) illustrating ASV-level microbial community composition across four *Ircinia* species. Points are colored by *Ircinia* species, and ellipses represent 95% confidence intervals.

**Figure S4** – The relative abundances of shared microbial ASVs between sponge hosts and *Risor ruber* (A) skin and (B) guts. *R. ruber* individuals are ordered by total length along the x-axis. The diagonals indicate shared microbial relative abundances with the sponge individual from which each fish was collected.

**Figure S5** – The relative abundances of shared microbial genera between sponge hosts and *Risor ruber* (A) skin and (B) guts. *Risor ruber* individuals are ordered by total length along the x-axis. The diagonals indicate shared microbial relative abundances with the sponge individual from which each fish was collected.

**Figure S6.** The relationship between R. ruber body size and the goby-sponge shared microbiome (genus level). *R. ruber* total length (mm) and the relative abundance of microbial genera shared between *R. ruber* skin (A) or gut (B) and sponge samples based on Bayesian beta regression models. Black points represent each individual fish–sponge pair. Lines depict posterior draws of the expected mean.
